# Knowledge, Attitude, and Practice of Postexposure Prophylaxis against HIV Infection among Healthcare Workers in Hiwot Fana Specialized University Hospital, Eastern Ethiopia

**DOI:** 10.1155/2019/7947086

**Published:** 2019-02-21

**Authors:** Endalkachew Mekonnen Eticha, Ashenafi Beru Gemeda

**Affiliations:** ^1^Department of Pharmacy, College of Health and Medical Science, Haramaya University, Harar, Ethiopia; ^2^Department of Clinical Nursing, College of Medicine and Health Science, Jigjiga University, Jigjiga, Ethiopia

## Abstract

**Background:**

Postexposure chemoprophylaxis can prevent human immunodeficiency virus (HIV) infection in risk health care workers; however routine adoption of these practices by the workers has been limited.

**Methods:**

A cross-sectional study was conducted on 311 health care workers of Hiwot Fana Specialized University Hospital between February and March 2016. Data was collected using a structured self-administered questionnaire and analysed using STATA 12.

**Results:**

In all, 83% of the participants had adequate knowledge of postexposure prophylaxis for HIV. All the respondents had heard about postexposure prophylaxis for HIV; however, only 37 (22.4%) workers know the definition of the postexposure prophylaxis. Among study participants, the majority of them, 272 (87.5%), knew the preferable time to initiate postexposure chemoprophylaxis. A significant number of the workers (43.4%) had an unfavorable attitude towards postexposure prophylaxis. Among 53 workers with a potential exposure to HIV, 38 (71.7%) took postexposure chemoprophylaxis and only 26 (44.8%) completed taking postexposure prophylaxis correctly.

**Conclusion:**

In all, most of the health care workers had adequate knowledge about postexposure prophylaxis against HIV/AIDS. The result shows that a significant number of individuals had a negative attitude and poor practice with regard to postexposure prophylaxis. Therefore, formal training that aims to improve attitudes and support to improve postexposure prophylaxis implementation and completion are needed. We would recommend the establishment of appropriate guidelines and the supply chain to ensure the availability of postexposure prophylaxis drugs for the protection of healthcare workers with potential high risk exposure to HIV.

## 1. Introduction

Acquired Immune Deficiency Syndrome (AIDS) is one of the most serious public health problems costing the lives of many people, particularly in sub-Saharan Africa where even health care workers (HCWs) are at affected and at risk [1, 2].

Occupational exposure to blood and body fluids is a serious concern for HCWs and presents a major risk for the transmission of infections such as Human immunodeficiency virus (HIV), Hepatitis B virus (HBV), and Hepatitis C virus (HCV) [3, 4]. According to World Health Organization (WHO) report of 2005, about 3 million percutaneous occupational exposures to blood or other bodily fluids occur in health care settings, the majority (90%) of which occurred in developing countries [5, 6]. Since the early 1990s, antiretroviral (ARV) drugs have been prescribed for postexposure prophylaxis (PEP) following occupational exposure to HIV for HCWs [7, 8]. PEP consists of administering 28 days of antiretroviral therapy (ART) as soon as possible up to 72 hours following high risk exposure to prevent establishment of HIV infection [9–11].

Although some studies reported favorable knowledge about PEP, there remains a knowledge gap among health care workers [12–14]. In Cameroon, 73.7% [15] and in Zimbabwe, 65% [16] of HCWs had poor knowledge. Similarly, one study had documented inadequate knowledge of PEP in up to 83.9% among HCWs in Ethiopia. Furthermore, among the exposed respondents, 81.6% did not use PEP; 33.8% of whom did not use PEP because of lack of information [2].

Currently, there is no data regarding PEP knowledge among HCWs in Harar, Eastern Ethiopia. Thus, this study was conducted to assess knowledge, attitude, and practice on occupational PEP to prevent HIV infection among health care workers of Hiwot Fana Specialized University Hospital (HFSUH), an eastern Ethiopia regional referral hospital.

## 2. Methodology

### 2.1. Study Period, Setting, and Participants

A hospital based cross-sectional study was conducted from February to March 2016 including 311 health care workers of HFSUH. The hospital is one of the six governmental teaching hospitals in Ethiopia that provides health care service to greater than 300,000 inhabitants. The hospital has 402 healthcare workers. The participants in this study were permanent employees of HFSUH who were routinely involved health care delivery during the study period.

### 2.2. Data Collection and Study Procedure

Data collection was conducted using a structured self-administered questionnaires prepared in English based on WHO postexposure prophylaxis guidelines and relevant published articles [4, 14, 17, 18]. Questions assessed knowledge, attitudes, and practices regarding PEP for HIV prevention. Ambiguous and unsuitable questions were modified after the pretest had been conducted. The validity of the developed questions was checked prior to finalizing the survey instrument. The pretest was conducted on 38 HCWs (10% of the study population) and they were excluded from participating in the main study.

### 2.3. Sample Size and Sampling Technique

A convenience sampling technique was employed. Of the 342 eligible workers, 311 HCWs were included in the study.

### 2.4. Scoring of Knowledge, Attitude, and Practice

Respondents who scored greater than or equal to 6 correct answers (75%) from 8 equitably scored knowledge questions were considered to have “adequate knowledge”. Similarly, respondents scored greater than or equal to 75% (6 out of 8 questions) of attitude questions were considered to have “positive attitude”. To determine the practice of respondents', those answered greater than or equal to six out of eight questions (≥ 75%) were considered as practicing PEP for HIV. The practices were evaluated based on correct responses on practices stipulated by guidelines at the time.

### 2.5. Data Analysis

Data was cleaned, coded, and entered into the STATA 12 software. The results were summarized in frequencies and percentages.

### 2.6. Ethical Consideration

Ethical clearance to conduct this study was secured and obtained from the ethical review board of college of Health and Medical Sciences of Haramaya University. Participants provided an explanation of the study aims and were included in the study after they provided their written and oral consent to the study. The confidentiality of the study participants was maintained by assigning unique study identifiers during data collection and analysis.

## 3. Results

### 3.1. Sociodemographic Characteristics

A total of 311 HCWs were involved, of which 157 (50.5%) were males and 154 (49.5%) were females. Most of the respondents, 283 (91%), were in the age group of 20 to 30 years with a mean age of 26.2 years. The majority of the participants were nurses (76.1%), of whom 41.8% had less than one year of experience as shown in Table 1.

### 3.2. Knowledge Level of the HCWs about PEP for HIV

In the current study, 258 (83%) of the participants had good knowledge about PEP for HIV. Although the entire respondents heard about PEP for HIV infection, only 37 (22.42) workers knew the meaning of PEP. The main source of the information was formal training, 127 (40.8%). The majority of the study participants knew the preferred time to initiate PEP, 272 (87.5%), and the maximum acceptable delay prior to initiating PEP for HIV, 266 (85.5%). As shown in Table 2, greater than 90% of the participants had adequate knowledge of the appropriate duration of PEP for HIV infection prevention after an accidental occupational exposure.

### 3.3. Attitude of the HCWs about PEP for HIV

Greater than half, 176 (56.6%), of the study participants had a positive attitude about PEP. The majority of the respondents, 288 (92.6%) and 250 (80.4%), agreed on the benefit of PEP and availability of PEP guidelines in their work place, respectively. The majority of individuals (72.0%) strongly believed that PEP can reduce the likelihood of acquiring HIV after being exposed and 51.8% of the respondents agreed that PEP prevents further infection. However, only 118 (37.9) of the participants believed that PEP should be indicated for any type of sharp object injuries. As indicated in Table 3, only 23 (7.4%) individuals had no trust in PEP effectiveness.

One in four workers (24.8%) does not agree that PEP is important if the exposure is not with patient blood of known HIV positive.

### 3.4. Practice Status of the HCWs towards PEP for HIV

Of the 53 (17.0%) individuals who had exposures for HIV risky conditions, 42 reported their exposure to program runner and 38 (71.7%) took PEP. However, 15 (28.3%) of the exposed respondents did not take PEP. Among the individuals who took PEP, 21/38 (55.3%) were exposed to blood from patients with known HIV infection, whereas the remaining 15/38 (39.5%) were exposed to blood from source patients of unknown HIV status.

Among all the respondents who took PEP, two individuals initiated outside of the ideal time-frame (after 72 hours). Ten (26.3%) individuals started within an hour of exposure. Of the 38 respondents who took PEP, 26 (68.4%) had completed taking PEP correctly, but the rest 12 of the individuals failed to complete PEP. The main reason for the discontinuation of PEP was found to be fear of the adverse effects (7 individuals) and doubt of its efficacy (4 individuals) as shown in Table 4.

## 4. Discussion

Adherence to the universal precaution guidelines is fundamental for the prevention of accidental acquisition of HIV infection in healthcare settings. Furthermore, the appropriate management of exposed individuals plays a crucial role in control and prevention of the infection [10, 17].

We found that, only 17% of the HCWs had poor knowledge of PEP for HIV. This is lower than similar studies from Gondar University Hospital (36.9%) [14], Nigeria (7-24%) [19, 20], and Cameroonian health district (26.3%) [15]. Greater than 90% of our study participants had completed their bachelor or medical degree; this higher level of education may explain the higher knowledge demonstrated by our participants.

In the current study, all the participants had heard about PEP for HIV, 40.48% via training. This level of awareness among our study participants was higher than similar studies from Gondar University Hospital (92.8%) [14], Hawassa University Hospital (67.1%) [21], and Tertiary Hospital of Nigeria (97%) [19].

Data from animal studies suggest that the efficacy of postexposure prophylaxis in preventing transmission is time dependent [19, 22–24], and every effort should be made to provide postexposure prophylaxis as soon as possible following exposure. Regarding timing and duration of PEP for HIV, 87.46% of the total respondents stated that PEP should be taken within one hour and 91.32% of them knew the correct duration of PEP against HIV/AIDS (28 days). A study conducted among Interns of a Medical College in West Bengal, India, indicated only 68.5% stated PEP should be started within an hour of exposure and only 46.9% conveyed appropriate duration of PEP (28 days) [25]. The difference might be due to differences in the work experience as greater than 50% of our participants had professional experience of greater than one year. In addition, our result showed greater awareness on timing of PEP among our HCWs than other studies from Uganda (22.3%) [26], Mumbai (64%) [27], and Gondar (50.8) [14].

The majority of our study participants had a positive attitude towards PEP. A study conducted at the Gondar University Hospital [14] indicated that 98.5% agreed on the importance of PEP for HIV, which is greater than our study (92.6%) and 69.5% agreed that PEP guidelines should be available in the hospital which is lower than our study (80.4%).

Of the 311 study subjects, 53 (17.2%) of the participants have been exposed to HIV risky conditions. This finding is less than the result found in the research done in the Jimma zone on government HCWs (68.50%) [2] and Gondar University Hospital (33.8%) [14]. Lower exposure of our study participants to risky conditions might be due to better knowledge in our study group. However, the number of HCWs that have ever been exposed to HIV risky conditions in our study is not considered low.

Among 38 HCWs on ART for PEP, 21 (55.3%) of them exposed to blood of known HIV positive patients, which is comparable to study from the Gondar University Hospital (57.1%) [14]. Even though 71.7% of the exposed respondents took PEP for HIV in this study, only 68.4% of them were able to complete the duration of prophylaxis which requires 28 days. The main reason for nonadherence of these individuals was fear of adverse effects. This indicated that it is lower than the findings of the study conducted among HCWs of governmental health institutions in Mekelle Town, Ethiopia (80.6%) [28]. However, a study conducted in Gujarat, India [29], showed that their respondents had better practice in this regard than our study participants in which more than 94% were able to complete the regimen. This fact alerts that the practice of PEP for HIV in this study area needs improvement. Reasons for the observed difference of findings between different research results might be due to the difference in the level of awareness among the different population, economic difference of the study population, and time difference of the studies.

## 5. Limitation of the Study

The expected limitations to this study are unwilling of HCWs in the hospital to participate in the study and the absence of HCWs at the time of data collection. Our statistical analysis was descriptive and we are unable to determine association of independent variables with the outcome. The convenience sampling technique was also the major limitation of the present study.

## 6. Conclusion

Most of HCWs have good knowledge about occupational risk of HIV/AIDS exposure and had a good attitude towards occupational risk of HIV infection. The findings of this study revealed the attitude and practice of HCWs towards PEP for HIV is inadequate. A significant proportion of HCWs have had exposure that would warrant the use of PEP. This compounded by low PEP completion rates shows that the practice of PEP for HIV in this study area needs improvement.

## Figures and Tables

**Table 1:** Sociodemographic characteristics of HCWs at Hiwot Fana Specialized University Hospital, 2016.

Variables	N (%)
*Age*	
20-30 year	283 (91.0)
31-40 year	26 (8.4)
41-50 year	1 (0.3)
>50	1 (0.3)
*Sex*	
Male	157 (50.5)
Female	154 (49.5)
*Marital status*	
Married	85 (27.3)
Single	223 (71.7)
Divorced	3 (1)
*Profession*	*N (*%)
Doctor	20 (6.4)
Medical Laboratory	22 (7.1)
Nurse	246 (76.1)
Health officers	3 (1)
Midwives	20 (6.4)
*Educational level*	
Diploma	9 (2.9)
First degree	282 (90.7)
MD	16 (5.1)
MSC or Specialist	4 (1.3)
*Work experience (in year)*	
<1	146 (47)
1-3	116 (37.3)
4-5	32 (10.3)
>5	17 (5.5)

**Table 2:** Knowledge about PEP for HIV among healthcare workers in Hiwot Fana Specialized University Hospital, 2016.

*Knowledge question*	N (%)
*Awareness of PEP*	
Yes	311 (100.0)
No	0

*Know the meaning of PEP*	
Yes	37 (22.4)
No	128 (77.6)

*Aware of the availability of PEP guideline in this hospital. *	
Yes	311 (100.0)
No	0

*Identify indication for PEP*	
When the source patient is at high risk for HIV *∗*	94 (30.2)
When the source patient is known to be HIV positive*∗*	161 (51.8)
When the HIV status of the source is unknown*∗*	36 (11.6)
For any needle stick injury in the work place	20 (6.4)

*The maximum delay to take PEP*	
12 hours	10 (3.2)
24 hours	19 (6.1)
48 hours	16 (5.1)
72 hours*∗*	266 (85.5)

*Preferable time to start PEP*	
Within an hour*∗*	272 (87.5)
After 6hours	20 (6.4)
After 12hours	10 (3.2)
After 72hour	9 (2.9)

*Duration of ART intake for PEP*	
For 28 days*∗*	284 (91.3)
For 40 days	20 (6.4)
For six months	5 (1.6)
For life time	2 (0.6)

*Know about the PEP guideline*	
Yes	198 (63.7)
No	113 (36.3)

*∗* indicates the correct answer from the range of choices per the WHO PEP guideline [7].

**Table 3:** Attitude of HCWs about PEP at Hiwot Fana Specialized University Hospital, 2016.

*Attitude question*	N (%)
*Think PEP is important*	
Yes	288 (92.6)
No	10 (3.2)
I am not sure	13 (4.2)

*PEP training is important for behavioural change*	
Yes	223 (71.7)
No	32 (10.3)
Neutral	56 (18.0)

*PEP guidelines should be available in the work area*	
Agree	250 (80.4)
Disagree	61 (19.6)

*PEP can reduce the likelihood of becoming HIV positive*	
Agree	224 (72.0)
Disagree I am not sure	22 (7.1) 65 (20.9)

*PEP prevents further infection*	
Agree	161 (51.8)
Disagree	150 (48.2)

*PEP should be indicated for any type of sharp injuries*	
Agree	118 (37.9)
Disagree	99 (31.8)
I am not sure	94 (30.2)

*PEP is not important if the exposure is not with blood of a patient with known HIV positive*	
Agree	155 (49.8)
Disagree	77 (24.8)
I am not sure	79 (25.4)

*PEP is effective for HIV prevention*	
Agree	219 (70.4)
Disagree	23 (7.4)
I am not sure	69 (22.2)

**Table 4:** Practice of PEP for HIV among HCW in Hiwot Fana Specialized University Hospital, 2016.

*Practice*	N (%)
*Occupational exposure to HIV risky conditions.*	
Yes	53 (17.0)
No	258 (83)

*Reported to the program Coordinator *	
Yes	42 (79.3)
No	11 (20.8)

*Reaction of HCWs toward HIV exposed individuals *	
Supportive and maintained confidentiality	33 (78.6)
Confidentiality was not maintained	6 (14.3)
Did not show concern about my accidental exposure	3 (7.1)

*PEP after exposure *	
Received	38 (71.7)
Not received	15(28.30)

*Reason for receiving PEP*	
Exposure to blood from known HIV positive patients	21 (55.3)
Exposure to blood from patient whose HIV status is unknown	15 (39.5)
Injury from any sharp object	2 (5.3)

*Time to initiate PEP after exposure*	
Within 1 hour	10 (26.3)
After 2-6 hours	16 (42.1)
After 6-10 hours	10 (26.3)
After 72 hour	2 (5.3)

*Duration of PEP*	
For 3 days	5 (13.2)
For 15 days	7 (18.4)
For 28 days	26 (68.4)

*Completed the prescribed ART for PEP*	
Yes	26 (68.4)
No	12 (31.6)

*Reason for discontinuation of the ART for PEP*	
Fear of adverse effects	7 (58.3)
Assuming that it was enough	1 (8.3)
Assuming that the drug was not effective	4 (33.3)

## Data Availability

The data used to support the findings of this study are available from the corresponding author upon request.
